# Comparative prospective study of 2 ovarian stimulation protocols in poor responders: effect on implantation rate and ongoing pregnancy

**DOI:** 10.1186/s12978-015-0039-2

**Published:** 2015-05-30

**Authors:** Philippe Merviel, Rosalie Cabry-Goubet, Emmanuelle Lourdel, Aviva Devaux, Naima Belhadri-Mansouri, Henri Copin, Moncef Benkhalifa

**Affiliations:** Department of Gynecology, Obstetrics and Reproductive Medicine, Amiens University Medical Center, 124 rue Camille Desmoulins, F-80054 Amiens cedex 1, France; Reproductive Biology and Medical Cytogenetics Department, University Hospital, 124 rue Camille Desmoulins, F-80054 Amiens cedex 1, France; School of Medicine, University of Picardie Jules Verne, Chemin du Thil, 80025 Amiens cedex 1, France

**Keywords:** Poor responder, IVF/ICSI, GnRH agonist, GnRH antagonist, Implantation and ongoing pregnancy

## Abstract

**Background:**

In patients treated with IVF, the incidence of poor ovarian response (POR) after ovarian stimulation varies from 9 to 25 %. However, at present, there are no clear guidelines for treating these poor responders. This study was designed to compare two different ovarian stimulation protocols and addresses future perspectives in the management of these unfortunate patients.

**Method:**

Four hundred and forty poor responders were studied during their second IVF cycle. They had all failed to become pregnant during their first IVF cycle where the long GnRH-agonist stimulation protocol (P1) was used. Patients were prospectively randomly assigned to 2 protocol groups (P2 or P3, 220 patients in each arm) at the start of ovarian stimulation according to the order of entry into the study including one patient per each stimulation protocols: The P2 group was treated with a contraceptive pill + flare-up GnRH-agonist protocol and the P3 group with the GnRH-antagonist protocol. The ovarian stimulation characteristics as well as the clinical and ongoing pregnancy rates were compared.

**Result(s):**

Although the numbers of embryos obtained and transferred were significantly higher with the P2 protocol, the implantation and ongoing pregnancy rates per transfer were the same in the two studied groups (8.9 % versus 14.6 % and 8.4 % versus 14.2 % for the P2 and P3 protocols, respectively). Good prognostic factors for ongoing pregnancy with both protocols were: a maternal age <36, no tobacco consumption, a total dose of gonadotropins injection <5000 IU and an endometrial thickness >10 mm.

**Conclusion(s):**

In poorly responding patients treated with IVF, the implantation and ongoing pregnancy rates per transfer were not significantly different between the two protocols studied: contraceptive pill + flare-up GnRH-agonist protocol and the GnRH-antagonist protocol. It is suggested that current strategies for the management of poor responders be reconsidered in the light of the potential contribution of age and the effect of life style changes on fertility potential. A customised policy of ovarian stimulation in these patients including mild stimulation protocols, sequential IVF cycles, oocytes-embryos freeze all protocols and blastocyst transfers after screening may improve the clinical outcome.

## Background

In routine IVF programs, the incidence of poor ovarian response (POR) after ovarian stimulation varies from 9 to 25 % of patients [1]. This poor response can be related to different causes such as age, endometriosis, ovarian surgery, genetics factors or may be iatrogenic. Although there is a lack of uniform definitions of poor response [2], the most common criteria used for diagnosis of poor responders is a low number of retrieved oocytes despite adequate ovarian stimulation [3].

Recently, an ESHRE consensus conference [4] published the “Bologna criteria” and defined the poor ovarian response by the presence of two of the following three features: (i) advanced maternal age (≥40 years) or any other risk factor for POR; (ii) a previous characterized POR cycle (≤3 oocytes with a conventional stimulation protocol); (iii) an abnormal ovarian reserve test (antral follicle count <5-7 follicles or AMH <0.5-1.1 ng/ml).

Several controlled ovarian hyper stimulation (COH) strategies have been described for treating poor responders, but at present, there are no clear guidelines for treating those patients. In practice many clinicians are increasing the daily gonadotropins dose (up to 450–600 IU/d), despite the lack of supporting evidence [5, 6]. The use of gonadotropin-releasing hormone agonist (Gn-RH agonist) long protocol in IVF leads to ovarian desensitization, resulting in a reduction in the number of mature follicles and the need to increase the dose of gonadotropins in poor responders [7, 8]. Other investigators have reported the advantages of the initial endogenous gonadotropin “flare” induced by Gn-RH agonist, enhancing the effect of exogenous gonadotropins [9, 10]. Given that significant increases in serum estradiol, androgen and progesterone levels have been noted with this protocol, Lindheim et al. [11] suggested that patients in whom Gn-RH agonist long protocol had failed might benefit from a combination of a pre-cycle oral contraceptive pill treatment and a micro dose Gn-RH agonist flare-up protocol. On the other hand, the introduction of GnRH antagonists (GnRH antagonist) have presented a hope for the poor responders in view of its immediate suppression of LH, the absence of flare-up effect, the reduction of the duration of stimulation and the dose of gonadotropins used. In poor responders, Fasoulitis et al. [12] using a GnRH antagonist protocol in poor responders reported a non-significant trend towards higher implantation and clinical pregnancies rates. However, other workers reported higher cancellation rates [13] or a reduced number of retrieved oocytes without a difference in the clinical pregnancy rate [14].

The aim of this prospective study was to compare two different protocols in poor responders who had one IVF failure when using a long GnRH agonist protocol, and to evaluate the perspectives and challenges facing poorly responding patients requiring IVF therapy.

## Materials and methods

Poor responders included in this study were defined as those patients from whom less than 4 mature oocytes were retrieved in the first stimulated IVF cycle using the Gn-RH agonist long protocol (P1 protocol). During this P1 protocol all patients were treated with triptorelin (0.1 mg/day SC, Decapeptyl®, Ipsen, Paris, France) from day 20 of the previous cycle, for 14 days. After achieving desensitization, the dose of triptorelin was diminished to (0.05 mg/day SC) and, at the same time, a fixed-dose of gonadotropin FSH or hMG (375 IU/day, SC) was started and injected daily until the day of hCG administration.

### Study design

**Fig. 1:**
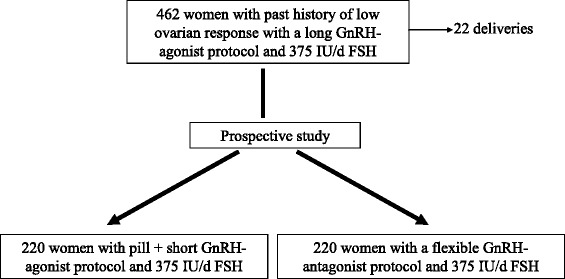
Chronology of the study

After failure of the first IVF cycle using the long GnRH agonist protocol, 440 women were prospectively randomized, after an interval of less than 4 months, to a prospective study comparing two other protocols (Fig. 1). The study design included two parallel groups with the same size using block randomization. The 440 patients were prospectively randomly assigned to 2 groups (220 patients per each) at the start of ovarian stimulation. The randomization of the two protocols (P2 or P3) was made according to the order of entry into the study including one patient per each stimulation protocols: Protocol 2, then 3, and so on. In this study, there was no control group. Protocol 1 is the basis of our definition of poor responders and the comparison is made between protocol 2 and protocol 3.

### Ethical considerations

The authors assert that all procedure contributing to this work comply with the ethical standards of the relevant (our) national and institutional committees on human experimentation and with the Helsinki declaration of 1975, as revised in 2008. All patients who participated in this study signed an informed consent after being informed about the terms and issues of the study.

### Stimulation protocols

Two stimulation protocols were compared:

1. A flare-up GnRHa protocol (P2 protocol): this group consisted of 220 women received a low dose oral contraceptive pill (Desolett®, MSD, Courbevoie, France) started on cycle day 1 of the previous cycle for 21 days. Three days after the end of the pill, triptorelin (0.025 mg/day) and a fixed-dose of gonadotropin FSH or hMG (375 IU/l) were administered until the day of hCG injection.
2. A multidose GnRH antagonist protocol (P3): the second group consisted of 220 women, in whom, on day 2 of a spontaneous cycle, gonadotropin stimulation was initiated at a fixed-dose of 375 IU/day. When the leading follicle reached 14 mm in mean diameter and/or plasma E2 exceeded 400 pg/ml and/or LH serum levels were >10 IU/l, an injection of 0.25 mg of cetrorelix GnRH antagonist (Cetrotide®, Merck Serono, Lyon, France) was administered SC daily until the day of hCG administration.

For controlled ovarian hyperstimulation (COH), we used urinary FSH (Fostimon®, Genevrier, Sophia-Antipolis, France), recombinant FSH (Gonal-F®, Merck Serono, Lyon, France; Puregon®, MSD, Courbevoie, France) or human menopausal gonadotropin hMG (Menopur®, Ferring SAS, St Prex, Switzerland), and as in the P1 protocol the FSH or hMG fixed dose was not adjusted according to the ovarian response. When at least three follicles reached a diameter of >17 mm, a dose of 250 μg of recombinant human chorionic gonadotropin (Ovitrelle®, Merck Serono, Lyon, France) was administered and trans-vaginal oocyte retrieval was performed 35–36 hours after hCG administration. Plasma E2 and plasma progesterone levels were measured on the day of hCG administration. Cycle cancellation was recommended when less than three mature follicles were observed. Intracytoplasmic sperm injection (ICSI) was performed only in cases with severe male factor or previous fertilization failure. For this procedure, the cumulus and corona radiata were exposed to 0.5 % hyaluronidase (Sigma Company, NY, USA) for 30s and removed mechanically under a dissecting microscope. After 18 h of incubation at 37 °C in a humidified atmosphere with 5 % CO_2_, oocytes were examined for the presence of two pronuclei, as a sign of fertilization. The embryos obtained were characterized into four grades depending on their morphological appearance (blastomere number and size, cytoplasmic fragmentation) as described by Terriou et al. [15]. Grade I/II embryos (high and good quality) were those with equal blastomeres and absence of or <20 % cytoplasmic fragmentation. A maximum of three embryos were transferred on day 2 or 3, using Frydman catheter (CCD, Paris, France). Other good-quality embryos were cryopreserved.

The luteal phase was supported by vaginal administration of 200 mg of micronized progesterone twice a day (Utrogestan®; Besins International, Paris, France), starting on the day of oocyte pick-up and continued for 20 days. Clinical pregnancy was diagnosed when serum β-hCG concentration was >1000 IU/l. Ongoing pregnancy was defined as the presence of an intrauterine gestational sac with cardiac activity 12 weeks after oocyte retrieval.

The demographic and medical data were recorded including the woman’s and man’s age, the type and duration of infertility, the woman’s body mass index (BMI) and any tobacco use. Day-3 follicle-stimulating hormone (FSH), luteinizing hormone (LH), 17β-estradiol (E2), inhibin B and anti-Müllerian hormone (AMH) serum levels were also measured.

### Statistical methods

Data were analyzed using Stat View for Windows, version 5.0.1 software (SAS Institute Inc., Cary, NC) and presented as mean +/− standard deviation (SD) or a standard number representing the total. The Student’s *t*-test or Mann–Whitney *U* test were used to compare continuous variables (mean ± SD), while the chi-square test or Fisher’s exact test were used to calculate the clinical and ongoing pregnancy rates. A *P* value < 0.05 was considered to be statistically significant with bilateral testing. Then mean values of clinical outcomes were evaluated to calculate the study power by post-hoc test using G*Power software (version 3.0.1). The power calculation showed that two samples of 220 patients resulted in a power of 80 % if the difference in percentage was 15 %. Multivariate logistic regression analysis was used to test the correlation between clinical variables on one hand and the occurrence of pregnancy on the other. Odds ratios (ORs) and 95 % confidence intervals (95 % CIs) were calculated separately for each factor. Confidence intervals exclusive of unity were considered to be significant.

## Results

Four hundred forty-two women, identified as “poor responders” after P1 protocol, underwent a second IVF treatment cycle between 2004 and 2011 at Amiens University hospital, and were randomized between P2 and P3 protocols. The results of the P1 protocol (n: 462) are reported in Table 1. Twenty two women had an ongoing pregnancy and were excluded from the prospective study. After randomization, the characteristics of the P2 and P3 protocol groups are shown in Table 2. There were no significant differences between the P2 and P3 groups in age, BMI, tobacco use, criteria of ovarian reserve, duration of infertility, type or indication of infertility. The stimulation cycle characteristics of the P2 and P3 groups are displayed in Table 3. Sixty-five percent of the cycles were ICSI procedures, with similar percentage in the two groups. Significant differences between P2 and P3 groups were noted in terms of the mean estradiol levels on the hCG administration day (1215 ± 350 vs. 712 ± 251 pg/ml; p < 0.001), embryos obtained (2.3 ± 0.5 vs. 2.1 ± 0.3; p < 0.001) and transferred (2.0 ± 0.2 vs. 1.9 ± 0.4; p < 0.01), but not in the ongoing pregnancy rate and the implantation rate per embryo transferred (p > 0.05). No twin or triplet gestations were seen in this study. The overall cancellation rate was the same for the two groups (P2: 19 % vs. P3: 23.1 %), despite a trend toward a higher cycle cancellation rate due to poor ovarian response in the P3 group (10.9 vs. 7.2 %; p > 0.05). The percentage of grade I/II embryo was not significantly different between the two groups (35.9 vs. 36.8 %). The fertilization and cleavage rates were the same in both groups. The clinical, ongoing and implantation rates were similar in P2 and P3 groups (17.9 % versus 14.6 %, 8.9 % versus 15.9 % and 14.2 % versus 8.4 %, respectively).

**Table 1:** Ovarian stimulation results in the P1 protocol

Number of cycles	462
Total FSH/hMG dose (IU)	4754 ± 684
Duration of stimulation (days)	12.6 ± 2.1
Mean estradiol levels on hCG day (pg/ml)	914 ± 307
Endometrial thickness (mm)	8.5 ± 1.6
Number of cancelled cycles (%)	75 (16.6)
Number of oocyte pick-ups	387
Number of oocytes retrieved (per pick-up)	1716 (4.5 ± 1.9)
Number of M2 oocytes retrieved	1437 (3.8 ± 2.8)
Number of oocytes fertilized	982
Number of embryos obtained	618 (1.6 ± 0.4)
Number of embryos transfer (per transfer)	329
Embryos per transfer	1.5 ± 0.3
Clinical pregnancy rate per transfer (%)	9.1
Ongoing pregnancy rate per transfer (%)	6.6
Implantation rate per embryo transferred (%)	6.0
Number of cycles with cryopreservation (%)	4.5
Number of cryopreserved embryos	35

**Table 2:** Patient characteristics in the P2 and P3 protocols

	P2	P3	*P* value*
Number of patients	220	220	
Number of cycles	220	220	
Age of woman (y)	38.0 ± 3.7	37.8 ± 3.1	
Age of man (y)	38.4 ± 5.6	38.0 ± 6.2	
BMI of woman (kg/m2)	24.6 ± 2.7	24.7 ± 2.8	
Woman with tobacco use (%)	38.1	38.6	
Day 3 FSH (IU/l)	9.7 ± 3.0	9.6 ± 3.1	
Day 3 LH (IU/l)	3.9 ± 2.5	4.2 ± 2.0	
Mean day 3 estradiol (± SD) (pg/ml)	70.5 (8.3)	69.3 (6.4)	
Mean day 3 inhibin B (± SD) (IU/l)	35.5 (5.2)	40.0 (5.1)	
Mean day 3 AMH (± SD) (ng/ml)	1.2 (0.4)	1.3 (0.7)	
% of patients with a total day 3–5 antral follicles < 6	27.2	25.9	
Duration of infertility (± SD) in y	4.6 (2.1)	4.7 (2.0)	
Primary infertility (%)	68.6	66.8	
Indications (%)			
Tubal	16.8	17.7	
Male	45.4	42.7	
Endometriosis stage I or II	12.2	10.0	
Mixed	12.2	14.0	
Unexplained	13.4	15.6	

*P* value*: All are not significant

**Table 3:** Ovarian stimulation results in the P2 and P3 COH protocols

	P2	P3
Number of cycles	220	220
Total FSH/hMG dose (IU)	4664 ± 605	4680 ± 641
Duration of stimulation (days)	11.8 ± 2.3	11.6 ± 2.7
Estradiol levels on hCG day (pg/ml)	1215 ± 350^a^	712 ± 251^b^
Progesterone levels on hCG day (ng/ml)	0.8 ± 0.2	0.7 ± 0.3
Endometrial thickness (mm)	8.7 ± 1.3	8.4 ± 1.2
No of oocyte pick-ups	204	196
No of oocytes retrieved (per pick-up)	1224 (6.0 ± 4.1)	1218 (6.2 ± 4.9)
No of M2 oocytes retrieved	894 (4.3 ± 3.7)	913 (4.6 ± 4.1)
No of oocytes fertilized	721	694
No of embryos obtained	487 (2.3 ± 0.5)^a^	426 (2.1 ± 0.3)^b^
Grade I/II embryos (%)	35.9	36.8
No of overall cancelled cycles (%)	42 (19.0)	51 (23.1)
No of embryos transfer	178	169
Embryos per transfer	2.1 ± 0.2^c^	1.9 ± 0.4^d^
Clinical pregnancy rate per transfer (%)	17.9	15.9
Ongoing pregnancy rate per transfer (%)	14.6	14.2
Implantation rate per embryo transferred (%)	8.9	8.4
No of cycles with cryopreservation (%)	15.7	9.4
No of cryopreserved embryos	69	43

^a-b^: significant difference at p < 0.001

^c-d^: *p* < 0.01

There was no significant difference between stimulation with FSH or hMG in the P2 and P3 protocols regarding the studied parameters. Prognostic factors of pregnancy during P2 and P3 protocols, obtained by a multivariate analysis, are summarized in Table 4. In contrast, no significant difference in clinical pregnancy rates was found in terms of man age, infertility duration, BMI, day 3 ovarian reserve evaluation (FSH, LH, estradiol, inhibin B and AMH), ovarian stimulation duration and estradiol level on hCG day.

**Table 4:** Prognostic factors in the P2 and P3 protocols correlated with occurrence of clinical pregnancy (in a multivariate analysis)

Factors	OR	95 % CIs	*p*
Female age < 36	2.39	1.45-3.34	<0.01
No woman tobacco use	3.05	1.62-4.48	< 0.02
Total dose of FSH/hMG < 5000 IU	1.77	1.11-2.93	< 0.05
Endometrial thickness > 10 mm	2.48	1.57-3.39	< 0.01

## Discussion

Definition of poor responders is still debated and many clinicians are using the Bologna criteria with or without adaptation according to personal experience [4, 16, 17]. In this study, we defined poor response as the retrieval of less than 4 mature oocytes with the long GnRH-agonist protocol, with a decrease of GnRH-agonist to 0.05 mg/day) after desensitization, as described by Feldberg et al. [7] and Olivennes et al. [8]. Using this definition, our current randomized study showed that, in those patients, the implantation and ongoing pregnancy rates per transfer were not significantly different between the contraceptive pill + flare-up GnRH-agonist P2 protocol and GnRH-antagonist P3 protocol.

The idea of using extremely low doses of GnRH-agonist in a flare regimen after oral contraceptive pretreatment (similar to our P2 protocol) has been reported by Surrey et al. [18] in 44 patients who had a poor response in a previous cycle stimulated with a long GnRH-agonist protocol (≤3 oocytes). Patients were divided into two groups, based on age (group 1: 15 women ≤ 39 years; group 2: 19 women ≥ 40 years). The cycle cancellation rate was dramatically reduced and the mean E2 level on hCG day was significantly increased with the pill + microdose flare-up GnRH-agonist protocol. The ongoing pregnancy rates per embryo transfer were 33 % in group 1 and 18.2 % in group 2. In contrast, three retrospective studies (Yakin et al. [19], Weissman et al. [20] and Detti et al. [21]) did not found any significant difference in clinical outcomes between the short flare-up microdose protocol compared to the long protocol with decreasing doses of the GnRH-agonist described by Faber et al. [22].

Several investigators have evaluated the role of GnRH-antagonist protocols in the treatment of poor responders. D’Amato et al. [23] reported in a study comparing the use of antagonists and the agonists in poor-responder women aged over 35. They found a lower cancellation cycle rate (4.7 % vs. 34.0 %, p < 0.0001), a higher number of oocytes (5.5 vs. 3.3, p < 0.0001) and embryo implantation rate (13.5 % vs. 7.6 %, p: non-significant) in the antagonist group. In another retrospective study, Berin et al. [24] found no significant difference in any IVF parameters and outcomes between the GnRH-antagonist (n: 68) and GnRH-agonist flare-up (n: 45) protocols. The only prospective, randomized trial investigating GnRH antagonist and microdose GnRH-agonist flare-up protocols in poor responders [14] reported no significant difference in outcomes, despite a trend towards higher implantation rates (15.0 vs 11.3 %) and ongoing pregnancy rates (21.0 vs 16.6 %) with the microdose GnRH-agonist flare-up protocol. However, despite an improvement in blastulation rate in the GnRH-antagonist protocol compared to the long GnRH-agonist protocol [25], there was no significant difference in the number and maturation rate of retrieved oocytes in both protocols, but only a slightly lower (but non-significant) implantation rate per transferred embryo with the GnRH-antagonist protocol [26, 27]. Moreover, the use of low-dose oral contraceptive from day 1 to 21 of the previous cycle did not result in any improvement in the final outcome, as shown in the studies of Malmusi et al. and Papras et al. [28, 29].

One of the major challenges in ovarian stimulation is to develop an optimal treatment regimen for patients who respond inadequately to initial treatment. In some cases, a suboptimal response may be due to under stimulation. In patients having shown a poor response to COH in their first cycle, Lashen et al. [6] investigated the value of increasing the gonadotropin dose in a subsequent cycle. The authors reported that patients who received 225 IU or more of gonadotropin daily in cycle 1 showed a similar poor response in their second cycle, despite the higher starting stimulation dose. Similarly, three studies [5, 30, 31] showed that increasing the hMG dose up to 450 IU per day in a second cycle neither increased the number of oocytes retrieved not improved the treatment cycle outcome, relative to a previous cycle with a lower hMG starting dose. In our study, although the initial dose of gonadotrophins was the same with the three protocols (375 IU/day), the numbers of embryos obtained for transfer were significantly higher with the P2 and P3 protocols compared to the first protocol (P1).

Our data support those reported in the literature and showing that, so far, no specific treatment improves the outcome in poor responders treated with IVF. Despite the consensus of Bologna, today there is no one clear definition of poor responder. In 2003 Toner et al. [32] reported that female age impacts oocyte quality and that both AMH and AFC profile affect the number of retrieved. To mitigate the negative effect of age, prophylactic oocyte cryopreservation can be proposed after ovarian stimulation for future use. This perspective is supported by advances made on vitrification [33]. However to reach pregnancy it will need between 12 to 15 oocytes [34] and even 22 to 50 depending on the woman’s age [35]. Today in France this perspective is not allowed but in the meantime we can offer mild stimulation to collect lower number of oocytes with better quality and giving acceptable number of embryo for vitrification [36]. This perspective is in line with the Baart et al. study [37] who found that that lower doses of gonadotropins are associated with embryos with lower aneuploidy rates and the Otsuki et al. [38] analysis which showed that high oestradiol levels can produce oocyte with vacuolated cytoplasm and reduce the chances of pregnancy. For poor responders, multiple mild stimulations followed by vitrification can increase the number of suitable embryo for transfer after thawing and improve clinical pregnancy. Our study showed that the improvement of pregnancy rate between protocol P1 and P2 or P3 is related to the number of suitable good embryos for transfer. In addition sequential transfer after vitrification on substituted or lightly stimulated cycles could improve implantation via better endometrial receptivity and ongoing pregnancy rate [39–41].

Finally, it seems that agonist and antagonist protocols involving high doses of gonadotropins are not beneficial in the management of poor responders and that new approaches need to be developed and proposed for these unfortunate patients consulting ART centers.

## Conclusion

In the present randomized study, we have found that the implantation and ongoing pregnancy rates per embryo transferred were not significantly different with the contraceptive pill + flare-up GnRH-a protocol compared to the multidose GnRH antagonist protocol. For these protocols, the prognostic factors for pregnancy were a maternal age <36, no tobacco consumption, a total FSH/hMG dose <5,000 IU and an endometrial thickness >10 mm. In the future we need to tailor our approach in the management of poor responders according to the woman’s age and life style. This can be achieved through a customised policy involving a mild stimulation strategy [42, 43], sequential IVF cycles, oocytes-embryos freeze all policy as well as blastocyst transfer after screening to improve the clinical outcome in those unfortunate patients.
